# Curcumin-Dichloroacetate Hybrid Molecule as an Antitumor Oral Drug against Multidrug-Resistant Advanced Bladder Cancers

**DOI:** 10.3390/cancers16173108

**Published:** 2024-09-08

**Authors:** Kunj Bihari Gupta, Truett L. Taylor, Siva S. Panda, Muthusamy Thangaraju, Bal. L. Lokeshwar

**Affiliations:** 1 Georgia Cancer Center, Augusta University, Augusta, GA 30912, USA; kugupta@augusta.edu (K.B.G.); trutaylor@augusta.edu (T.L.T.); 2 Department of Chemistry and Biochemistry, College of Science and Mathematics, Augusta University, Augusta, GA 30912, USA; sipanda@augusta.edu; 3 Department of Biochemistry and Molecular Biology, Medical College of Georgia, Augusta University, Augusta, GA 30912, USA; mthangaraju@augusta.edu

**Keywords:** chemically modified curcumin-2 (CMC-2), urinary bladder cancer (BCa), chemotherapy-drug-resistant BCa, neoadjuvant chemotherapy, oxidative stress, apoptosis

## Abstract

**Simple Summary:**

Advanced urinary bladder cancer (BCa) is characterized by rapid progression and development of therapy resistance. Despite all the available therapies, the five-year survival of metastatic BCa is a dismal 8% or lower. Many conjugates and additives of the natural polyphenol curcumin were developed and tested for potential therapeutic for cancers, with little success in vivo due to their rapid elimination and poor tissue penetrance. We tested a novel molecular hybrid CMC-2 composed of a curcumin scaffold conjugated to two dichloroacetate and glycine molecules. CMC-2 showed significantly higher antiproliferative and anti-survival activities against BCa than its parent compounds. Further, it was equally effective against chemotherapy-naïve and multidrug-resistant BCas (MDR-BCa). When tested in vivo using BCa xenograft in athymic mice, CMC-2 significantly delayed tumor growth without systemic toxicity. The mechanism of anticancer activity of CMC-2 was the induction of excessive oxidative stress in the cancer cells, leading to apoptotic cell death. These results show the potential of CMC-2 as a nontoxic oral treatment for high-grade bladder cancers.

**Abstract:**

Tumor cells produce excessive reactive oxygen species (ROS) but cannot detoxify ROS if they are due to an external agent. An agent that produces toxic levels of ROS, specifically in tumor cells, could be an effective anticancer drug. CMC-2 is a molecular hybrid of the bioactive polyphenol curcumin conjugated to dichloroacetate (DCA) via a glycine bridge. The CMC-2 was tested for its cytotoxic antitumor activities and killed both naïve and multidrug-resistant (MDR) bladder cancer (BCa) cells with equal potency (<1.0 µM); CMC-2 was about 10–15 folds more potent than curcumin or DCA. Growth of human BCa xenograft in mice was reduced by >50% by oral gavage of 50 mg/kg of CMC-2 without recognizable systemic toxicity. Doses that used curcumin or DCA showed minimum antitumor effects. In vitro, the toxicity of CMC-2 in both naïve and MDR cells depended on increased intracellular ROS in tumor cells but not in normal cells at comparable doses. Increased ROS caused the permeabilization of mitochondria and induced apoptosis. Further, adding N-Acetyl cysteine (NAC), a hydroxyl radical scavenger, abolished excessive ROS production and CMC-2’s cytotoxicity. The lack of systemic toxicity, equal potency against chemotherapy -naïve and resistant tumors, and oral bioavailability establish the potential of CMC-2 as a potent drug against bladder cancers.

## 1. Introduction

Curcumin (Cur; Diferuloylmethane) is a principal constituent of turmeric tuber, an edible spice. Cur is an antioxidant, antimicrobial, and anti-inflammatory natural product [1]. Oral or parenteral Cur infusion is well tolerated in humans with minimal systemic toxicity. The European Food Safety Authority (EFSA) has reported no systemic toxicity in humans at a daily intake of up to 3 mg/kg of body weight [2]. Cur has been tested for its therapeutic potential against many diseases, including malignant cancers of the breast, bladder, colon, pancreas, and prostate [3,4,5]. Although most studies conducted in vitro against tumor cells show a promising role of Cur in cancer treatment, its potential as an antitumor agent has been largely limited to experimental systems. Its use as a chemo-dietary supplement has been limited due to its poor systemic bioavailability via the oral route and its rapid elimination from the gut [6].

Dichloroacetate (DCA) is a potent inhibitor of lactic acidosis caused by cardiomyopathy. It inhibits pyruvate dehydrogenase kinase-2 (PDK-2) and alters metabolic pathways [7]. DCA increases mitochondrial ROS in tumor cells, activates K+ channels, decreases mitochondrial transmembrane potentials (ΔΨm), etc. [8]. Since DCA targets the metabolism of cancer cells, it has been tested for potential cancer treatment. Various studies have shown that DCA is cytotoxic to BCa cells and induces apoptosis [9]. Studies show that DCA’s efficacy significantly increases when combined with cisplatin in BCa [10,11]. DCA has not been approved for human use for any condition due to its rapid elimination and systemic toxicity. However, a combination of DCA, a safer glycolysis inhibitor, and a hybrid molecule of Cur might incorporate the biological functions of both molecules.

Several chemical modifications, formulations, additives, and analogs of Cur have been reported for their anticancer properties against several cancers [12,13]. Recently, we reported the design and synthesis of conjugates of curcumin (CMC-2) using a molecular hybridization approach [14]. This compound is the conjugate of Cur and DCA, which is linked with glycine molecules. The rationale is that this hybrid molecule may have higher bioavailability and antitumor activity than DCA and Cur alone. In the present study, we investigated the cytotoxic and antitumor properties and molecular mechanisms of cell death induced by CMC-2 against BCa. We evaluated the potency of CMC-2 in both in vitro and in vivo settings, using naïve (WT) and cells that had become multidrug-resistant to gemcitabine, cisplatin, and their combination. Our results show significant potential for the hybrid compound, CMC-2, to become a key player in future therapeutic developments.

## 2. Materials and Methods

### 2.1. Reagents and Chemicals

All reagents were purchased from laboratory suppliers based in the USA, and details on specialty chemicals and detection kits used in this study are listed below in Table 1.

### 2.2. Synthesis of CMC-2

**Figure 1:**
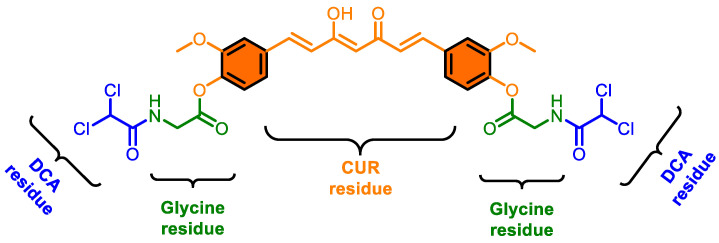
Chemical structure of CMC-2.

CMC-2 (Figure 1) was created from the molecular hybridization of curcumin and dichloroacetate (DCA) using glycine as a linker. The final chemical formula for CMC-2 is C_29_H_26_C_l4_N_2_O_10_, and its IUPAC name is ((1E,3Z,6E)-3-Hydroxy-5-oxohepta-1,3,6-triene-1,7-diyl) bis(2-methoxy-4,1-phenylene) bis(2-(2,2-dichloroacetamido) acetate); its other chemical name is (2,2-dichloroacetyl)glycine-acylated curcumin diester, and it has a molecular weight of 704.331 g/mole. Details of the synthesis of CMC-2 and its chemical and spectral characterization were reported in an earlier publication [14].

### 2.3. Procedures Related to Cell Culture, Maintenance, and Cell Line Authentication

The human bladder cancer cell lines CRL-1472 (HT-1376), HTB-9 (5637), and HTB-4 (T24) were purchased from the American Type-culture Collection (ATCC.org). The BCa cell line 253J was a gift from Dr. Colin N.P. Dinney (University of Texas, MD Anderson Cancer Center, Houston, TX). The non-tumorigenic bladder epithelial cell line UROtsa, established by an SV40 large T-antigen transfection, was a kind gift from Dr. Don Sens (University of Virginia, Charlottesville, VA) [15]. All cell lines were routinely cultured in a Complete Medium [RPMI1640 (Corning; Manassas, VA, USA) basal medium plus 10% fetal bovine serum (FBS, Biowest; Bradenton, FL, USA) and 10 mg/L gentamycin sulfate (IBI scientific, Dubuque, IA, USA)] and maintained as reported before [16]. Cell lines were genetically authenticated by Cell Line Authentication Services from Lab Corp Inc. (Burlington, NC, USA) and periodically tested against mycoplasma contaminants using a kit (Lonza Inc., Greenwood, SC, USA).

We selected 5637 cells that were stably resistant to gemcitabine (G), cisplatin (C), and G+C by growing the 5637 cells in increasing concentrations of these drugs in an antibiotic-free culture media, as described before [17].

### 2.4. Establishing Stable Multidrug-Resistant BCa Cell Lines

We recently reported a detailed characterization of laboratory-generated stable multidrug-resistant cell lines [17]. Briefly, a human BCa cell line 5637 was selected for survival in the presence of cisplatin (C), gemcitabine (G), and a combination of G&C for 26+ passages. As reported, the selected 5637 GemR, 5637 CisR, and 5637 G+C R cells are 8× to 10× more resistant to chemotherapy drugs than the unexposed 5637 naïve cells.

### 2.5. Cell Viability Assessment

Since the BCa cells form adherent monolayers in multi-well plastic culture plates, we tested their survival potential using a colorimetric and colony formation assay [17]. After seeding ~2000 cells/well in 96-well clusters for 24 h, various drugs were added along with the drug diluent (0.2% DMSO) and were cultured for 72 h in a humidified 37 °C incubator. We added 3-(4,5-dimethylthiazol-2-yl)-2,5-diphenyltetrazolium bromide (MTT) at a final 100 µg/mL concentration for three hours. At the end of three hours, the viable cells reduced MTT into intracellular formazan crystals were solubilized in situ with dimethyl sulfoxide, and the resulting colored solution was read at 575/650 nm in a multi-well-plate reader to determine their optical density (OD). The percentage of live cells was calculated from the ratio of OD values of treated culture wells divided by the OD of untreated control culture wells X100 [18,19].

### 2.6. Colony Forming Assay

The human naïve BC (HT1376 & 5637) and dual drug-resistant 5637 G+C R BC cells were cultured in 6-well plates at a density of ~2000 cells per well in triplicate. We treated cells with several doses of DCA, Cur, and CMC-2 for 7–10 days, with routine changing of the media with drugs, and the cells were allowed to form clonal colonies. The fixed and crystal violet-stained cell colonies were manually counted and expressed as a percentage of the colonies in untreated (or vehicle-only) cultures [17].

### 2.7. Estimating Reactive Oxygen Species (ROS)

The total reactive oxygen species (ROS) generated following treatment with drug-diluent (vehicle) and several doses of DCA, Cur, and CMC-2, with and without N-acetyl cysteine (NAC), were measured using a probe of 2′,7′-Dichlorodihydrofluorescein diacetate (DCFDA). Cells were cultured in 96-well plates at a density of 5000 cells per well overnight and then were treated with the appropriate concentration of drugs until an indicated time. After the incubation with drugs or control, culture wells were washed with PBS and incubated with DCFDA (10 μM) for 30 min at 37 °C. Subsequently, the unbound dye was removed, the wells were rewashed with PBS, and the fluorescence intensity of each well was recorded using a microplate reader (BioTek Synergy HTX Multimode Reader, Agilent, Santa Clara, CA, USA) at an excitation level of 485/20 and emission of 528/20. Data are represented as a percentage change from the drug-free, diluent only wells (control) [20].

The mitochondrial ROS (mt-ROS) levels generated in cells were measured using a dye MitoSOX™ with a procedure suggested by the vendor (Thermo-Fisher Inc., Eugene, OR). The fluorescence intensity of each well was recorded using a microplate reader (BioTek Synergy HTX, Agilent, Santa Clara, CA, USA), and the data are represented as a percentage change from the drug-free, diluent-only wells (control).

### 2.8. Estimation of Mitochondrial Trans-Membrane Potential (∆ψM)

We analyzed the changes in mitochondrial transmembrane potential in the BCa cells using a JC-1 dye J-aggregation assay by flow cytometry. As reported previously [17], we prepared single-cell suspensions from drug-treated cell cultures and incubated them with 10 µM of JC-1 for 30 min at 37 °C. JC-1, a green dye, forms red J-aggregates upon entry into the mitochondrial membrane due to its transmembrane potential but remains a green-fluorescent monomer in permeabilized mitochondria. We analyzed labeled cell suspension using flow cytometry to estimate the relative fraction of cells containing polymerized JC-1 aggregates that exhibit red and those with monomers only exhibiting green fluorescence in drug-treated and control cells.

### 2.9. Measuring Changes in Cell Cycle Phase Fractions

We plated 2 × 10^5^ cells/well in 6-well plates for 18 h to 20 h before adding diluted drugs to their required final dose; they were then incubated for the next 24 h. Nuclei suspension of cultured cells was prepared by suspending the cells in a hypotonic solution [50 mg Na-citrate, 15 µL of IGEPAL CA-630, and 0.1 mg/mL propidium iodide (PI)]. Nuclei suspension stained with PI was fractionated on an analytical flow cytometer to determine the percentage of cells at various cell cycle phases based on the relative DNA content. Data acquired on the NovoCyte Quanteon Flow Cytometer (Agilent, Santa Clara, CA, USA) were analyzed using NovoExpress software (version 1.6.1) [21].

### 2.10. Detection of Apoptosis

Drug-induced apoptosis of BCa cells was detected by labeling the cells with FITC-Annexin V, which detects externalized phosphatidyl serine on the plasma membrane, a commonly used test for detecting apoptotic activity [22]. Annexin binding and quantifying various levels of apoptotic activity were carried out using a ready-to-use FITC-Annexin V labeling kit as per the manufacturer’s instructions. Intact cell suspension labeled with FITC-Annexin V was run on the NovoCyte Quanteon flow cytometer, and data were acquired in list mode. Acquired data were analyzed using NovoExpress software to quantify levels of bound FITC-Annexin and apoptosis. The software allowed us to estimate the binding of FITC-Annexin to the varying stages of apoptosis [17].

### 2.11. Protein Immunoblotting (Western Blotting)

Cell lysates were prepared in RIPA buffer from cultures set up in 6-well plates and incubated with drugs for 24 h. Total protein content was quantified with the Pierce™ Micro BCA Protein Assay Kit (Thermo Scintific, Rockford, IL, USA). Volumes of cell lysates corresponding to 20 µg total protein/sample were mixed with SDS-Gel Sample buffer. They were then denatured and reduced by boiling them in 0.1 M DTT. Denatured samples were fractionated by SDS-PAGE and blotted onto a PVDF membrane (Merck Millipore, Tullagreen, Ireland #IPVH00010). Blots containing resolved proteins were immunoblotted as described before [17]. The list of antibodies and their dilutions used to detect various blotted proteins are listed below in Table 2.

### 2.12. In Vivo Studies to Test Antitumor Activities of DCA, Cur, and CMC-2

Xenograft studies in athymic mice determined the potential antitumor activities of the indicated drugs. All in vivo studies on mice were initiated and conducted using protocols approved by the official Augusta University’s Institutional Animal Care and Use Committee (IACUC, Augusta University; Protocol No.2016-0803). A statistical power analysis determined a group size of five per treatment with 80% power and a small error coefficient (α = 0.05), assuming a 100% tumor take rate with the BCa cell line HT-1376 cells. Male and female FOXN1 athymic mice (Crl: NU(NCr)-Foxn1^nu^) were purchased from Charles River Laboratories (Durham, NC, USA). Tumors were generated in mice by subcutaneous injection of ~1 × 10^6^ HT1376 cells suspended in 50% pathogen-free basement membrane extract (BME, R&D Systems) into the left or right flank of 8-week-old mice. Tumor growth was monitored by palpating the injected area twice weekly. The volume of palpable tumors was measured over time manually with a hand-held caliper, as described before [17]. Treatment began when all animals developed palpable tumors. Mice were gavaged daily with drugs (50 mg/kg) suspended by sonication in a solution of 2% aqueous 4-hydroxy methyl cellulose with 1% sucrose and 0.02% Tween-80 (vehicle), which was were prepared fresh each day. The potential toxicity of the drugs in the experimental groups was assessed by visual observation at the time of tumor measurement and by recording the body weight of all mice individually. Once the tumors in the control group averaged ≥ 500 mm^3^, mice were euthanized, and the tumors were dissected and weighed. Tumor tissues were fixed in formalin and processed for histology.

### 2.13. Statistical Analysis

All in vitro treatments and measurements were performed on triplicate samples, and all experiments were repeated at least three times. All statistical analyses were performed using GraphPad Prism 9 (GraphPad Inc., San Diego, CA, USA, Version 10.2.2). Unless indicated, data presented are mean ± S.D for control and experimental samples. We used the Student’s *t*-test or ANOVA for all statistical validations of observed data. Data with *p* ≥ 0.05 were rejected as random observations. A value of *p* < 0.05 for the null hypothesis was considered statistically significant.

## 3. Results

### 3.1. Cytotoxicity of DCA, Cur, and CMC-2 on BCa Cells

The cytotoxicity of DCA, Cur, and CMC-2 on chemo-sensitive and -resistant BCa cells and non-transformed, non-tumorigenic UROtsa cells was investigated using MTT and colony formation assays.

**Figure 2:**
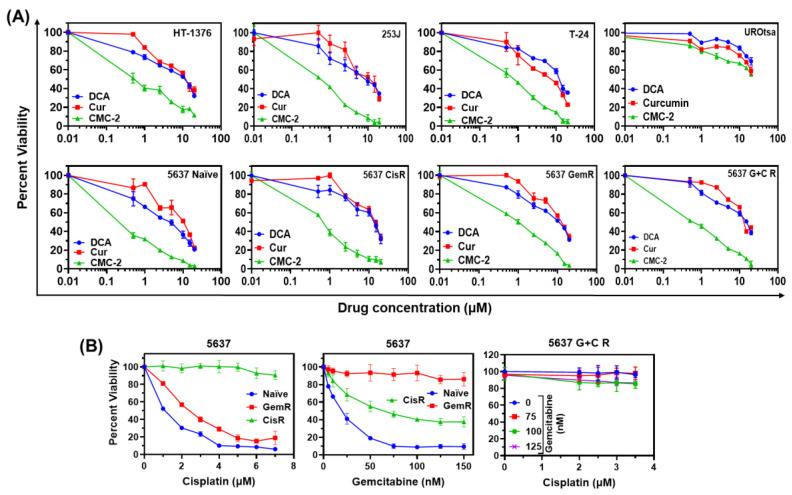
Cur, DCA, and CMC-2 are cytotoxic to BCa cells but were relatively nontoxic to non-transformed bladder epithelial cells, UROtsa: (**A**). Viability of tumorigenic, chemo-sensitive (HT-1376, 253J, T24, and 5637), chemo-resistant (5637 GemR, 5637 CisR, and 5637 G+C R) and non-tumorigenic (UROtsa) human BCa cell lines, treated with drugs or drug-diluent (control). (**B**). The viability of chemo-resistant human BCa, 5637 CisR, 5637 GemR, and 5637 G+C R cells as percent of untreated control. The MTT reduction assay was performed after 72 h of respective drug treatment. Results are expressed as mean of percent cell viability ± standard deviation (SD) (*n* = 4). Except for the non-tumorigenic UROtsa cells, CMC-2 was significantly more cytotoxic than Cur or DCA (*p* ≤ 0.05).

As shown in Figure 2A., the cytotoxic activity of the tested drug varied greatly. All three compounds (Cur, CMC-2, and DCA) were significantly more cytotoxic to BCa cells than their activity against URotsa cells. As shown in Table 3, the concentration of the drug at which survival was ≥50% (IC50) for DCA ranged from 7.99 μM to 12.65 μM, and the IC50 for Cur ranged from 7.91 μM to 12.12 μM. Whereas for CMC-2, the IC50 value was less than 1 μM (0.48 μM to 0.93 μM), showing that CMC-2 is significantly more potent (>15×) compared to DCA and Cur.

In the multidrug-resistant BCa cells (single drug-resistant 5637 GemR and 5637 CisR, and dual drug-resistant 5637 G+C R), the IC50 was elevated for DCA and Cur as compared to the chemo-sensitive BCa cells (5637 naïve). However, the cytotoxicity of CMC-2 in chemo-sensitive and multidrug-resistant BCa cells was still at sub-micromolar dose, showing that CMC-2 is effective against chemo-sensitive and multidrug-resistant BCa cells. CMC-2 was minimally toxic to UROtsa cells at these tested drug doses. (Figure 2A, and Table 3).

**Figure 3:**
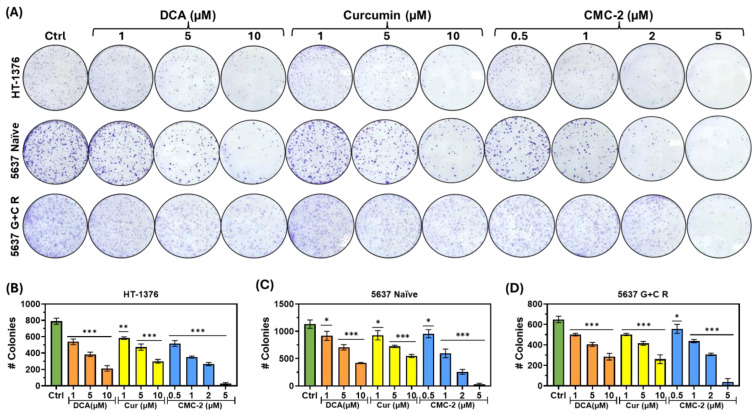
Clonal survival of human BCa cells exposed to DCA, Cur, and CMC-2: (**A**) Colonies formed by surviving cells after treatment with several doses of CMC-2, Cur, and DCA for three days that were then allowed to form colonies of >16 cells in the absence of drugs for next 4–7 days. (**B**–**D**). Graphs represent the mean of number of surviving colonies ± standard deviation (*n* = 3) for each cell type: (**B**) HT-1376 cells, (**C**) 5637 naïve, and (**D**) 5637 G+C R. Paired *t*-tests were used to determine the significance of difference between control and the exposed cells. * *p* ≤ 0.05, ** *p* ≤ 0.01, *** *p* ≤ 0.005.

MTT assays detect metabolic activity but are not accurate in distinguishing the cytostatic and cytotoxic effects of the drugs. The potential of these drugs to diminish the clonogenic survival potential was quantified using a colony formation assay. As shown in Figure 3A–D, the colony size and numbers declined significantly in chemo-sensitive cells (HT-1376 and 5637 naïve) and chemo-resistant cells (5637 G+C R) in a dose-dependent fashion, compared to the untreated control. These results demonstrate that all three drugs decrease colony-forming potential and that CMC-2 was significantly more potent than Cur and DCA. Colony assay results supporting the drug resistance of different 5637 cell lines (5637 GemR, 5637 CisR, and 5637 G+C R) are shown in Supplementary Figure S1.

### 3.2. Mechanism of DCA, Cur, and CMC-2 Cytotoxicity

Cur and other polyphenols exert their cytotoxic effects at the cellular level by multiple mechanisms. One mechanism is disrupting the mitochondrial oxidative status and the generation of ROS. Excessive ROS may lead to apoptosis via the intrinsic apoptotic mechanism mediated by changes in mitochondrial membrane permeability and transmembrane potential [23].

**Figure 4:**
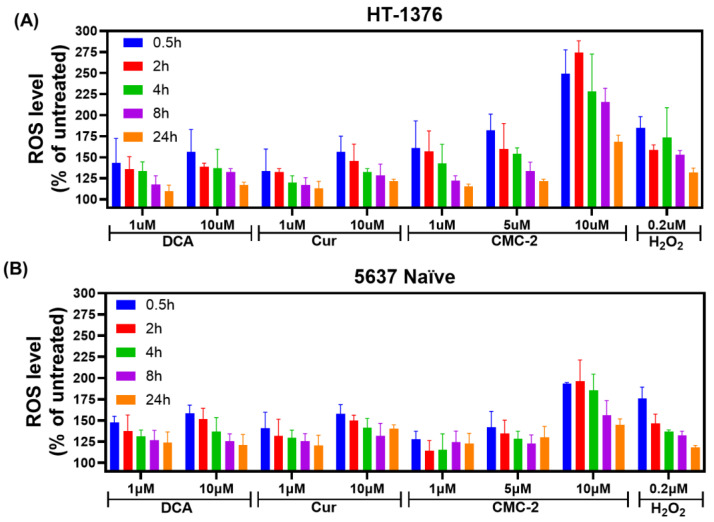
Estimating oxidative stress: reactive oxygen species (ROS) produced in (**A**) HT-1376 and (**B**) 5637 naïve cells after treatment with DCA, Cur, and CMC-2 for various periods, as estimated using a DCFDA probe. The relative fluorescence unit (RFU) was converted to the percent ROS value. Data represented here are means of ROS produced ± standard deviation (*n* = 4) after subtracting the ROS values generated in the untreated cells. Cells treated with H_2_O_2_ (0.2 µM) served as a positive control. ROS levels were significantly higher (*p* ≤ 0.05) in CMC-2-treated cells at the dose level of 10 µM of DCA, Cur, and CMC-2 for all treatment periods in both cell lines.

We investigated whether CMC-2, as compared to DCA and Cur, increased ROS generation in BCa cells. As reported before, we used the cell-permeable fluorescence probe dichloro dihydrofluorescein diacetate (DCFDA) to determine the levels of ROS in the cells. DCFDA is converted to dichlorofluorescein by intracellular deacetylase and oxidized to fluorescein by ROS, accumulating intracellularly. As shown in Figure 4, based on the fluorometric reading, the ROS levels were higher in drug-treated cells than in untreated cells (control). They were comparable to those generated by incubating with 0.02 µM of H_2_O_2_.

Further, the fluorescence intensity expressed in the control samples was significantly higher in CMC-2-treated cells than in DCA and Cur. The dose-dependent ROS increase was nearly identical in HT-1376 and 5637 naïve cells. The level of total ROS production tended to increase as the dose of these drugs increased, indicating total ROS production is dependent on drug dose. Moreover, the ROS levels rose immediately after exposing them to all three drugs individually, rising to the highest levels within 30 min of exposure. Although the fluorescence reading, which indicates ROS generation, declined with time, it was consistently high compared to untreated cultures after 24 h. This finding demonstrates that even though ROS has a short lifespan, the production of ROS continues due to the presence of these drugs.

**Figure 5:**
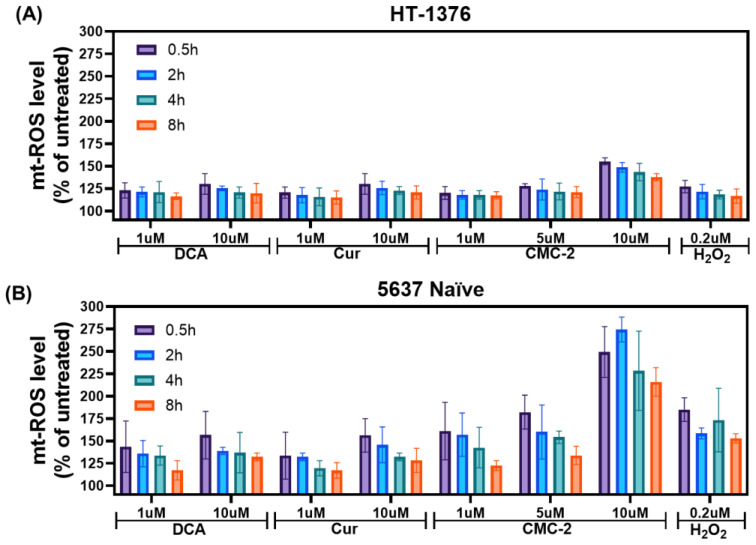
Estimating mitochondrial ROS (mt-ROS): the mt-ROS produced in the (**A**) HT-1376 and (**B**). 5637 naïve cells after the treatment of several doses of DCA, Cur, and CMC-2 exposed for several periods, as estimated using MitoSOX. The relative fluorescence unit (RFU) was converted to percent mt-ROS. Data represented here are means of mt-ROS produced ± standard deviation (*n* = 4) after subtracting the ROS values generated in the untreated cells. Cells treated with H_2_O_2_ (0.2 µM) served as a positive control. mt-ROS levels were significantly higher (*p* ≤ 0.05) in CMC-2-treated cells at the dose level of 10 µM of DCA, Cur, and CMC-2 for all treatment periods in both cell lines.

A leaky electron transport system is the primary source of mitochondria-specific superoxide and ROS (mt-ROS) production. As stress increases, the chances of this leakiness lead to higher mt-ROS. The mt-ROS production levels in HT-1376 and 5637 naïve cells after treatment with DCA, Cur, and CMC-2 were estimated using the molecular probe, fluorescence dye, and MitoSOX. We found the levels of mt-ROS were significantly higher in treated cells as compared with untreated BCa cells (Figure 5). Further, mtROS was considerably higher in CMC-2-treated cells compared to the other two drugs and even higher than that observed in H_2_O_2_-treated cells.

**Figure 6:**
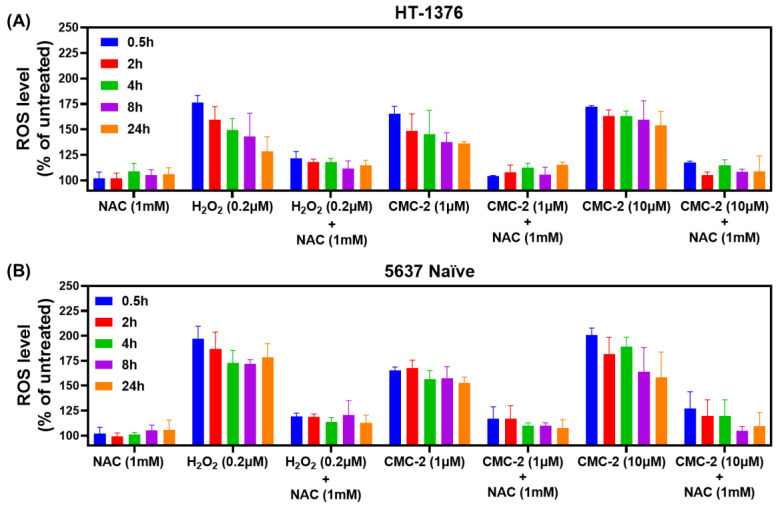
NAC abolishes ROS generation in CMC-2-treated cells: the level of ROS produced in (**A**) HT-1376, (**B**) 5637 naïve cells, after treatment with several doses of CMC-2 with and without N-acetyl-L-cysteine (NAC, 1 mM), was estimated using a probe DCFDA for 30 min, and intracellular fluorescence was measured. The relative fluorescence unit (RFU) was converted to the percent ROS value. Data represented here are means of ROS produced ± standard deviation (*n* = 4) after subtracting the ROS values generated in the untreated cells. Cells treated with H_2_O_2_ (0.2 µM) served as a positive control.

N-acetyl cysteine (NAC) is a well-known free radical scavenger. It inhibits the increase in intracellular ROS levels by promoting glutathione synthesis [24]. We treated the HT-1376 and 5637 naïve cells with CMC-2 and without NAC. We observed that NAC itself did not change ROS status in HT-1376 or 5637 naïve cells, but when these cells were exposed to NAC and DCA, Cur, or CMC-2, the increase in ROS was abolished (Figure 6).

**Figure 7:**
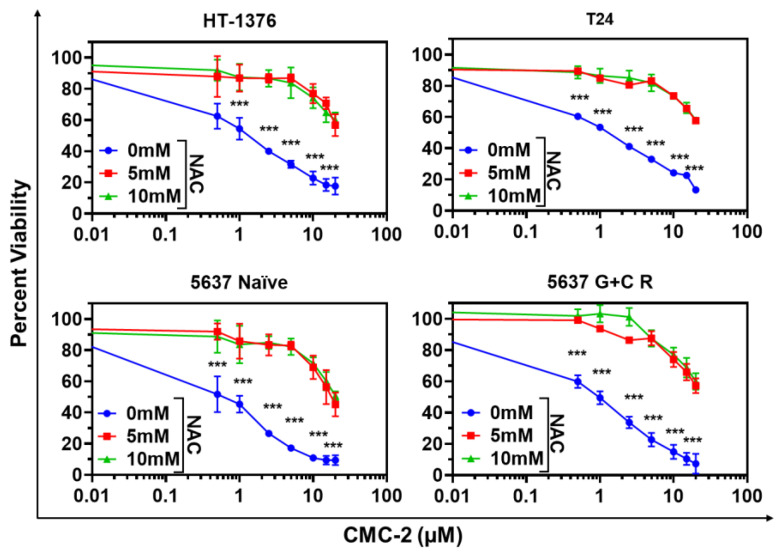
NAC abolishes CMC-2-induced cytotoxicity. MTT assay-based cellular viability was estimated in HT-1376, T24, 5637 naïve, and 5637 G+C R cells after treatment with several concentrations of CMC-2, NAC alone, and in combination. The percent cell viability was calculated relative to the untreated control cells, and results are expressed as the mean of percent cell viability ± standard deviation (*n* = 4) for each treatment. Paired *t*-tests were used to determine the significance of the difference in the cellular viability between CMC-2 and the combination treatment group. *** *p* ≤ 0.005.

The results of these experiments suggested that we test whether increased ROS is the primary mechanism of cytotoxicity of these drugs. We tested this hypothesis by estimating the viability of cells treated with these compounds and several NAC doses. As shown in Figure 7, NAC alone was not toxic to any BC cells at ≤10 mM. However, it significantly blocked CMC-2 toxicity in all treated cells.

### 3.3. Mitochondrial Transmembrane Potential (MTP; ψM) Is Significantly Decreased in CMC-2-Treated BC Cells

**Figure 8:**
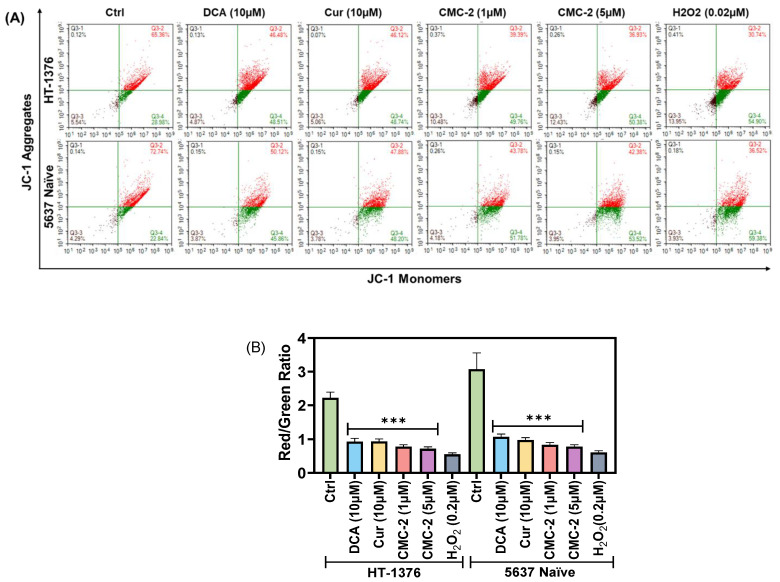
CMC-2 reduces mitochondrial transmembrane potential (ψM). Levels of ψM in human BC HT-1376 and 5637 naïve cells were estimated by JC-1 aggregates using flow cytometry after a 24 h treatment with DCA, Cur, and CMC-2. (**A**). Distribution of cells based on red and green fluorescence channels. (**B**). Bar graph demonstrating the red/green ratio of JC-1 fluorescence. The red/green ratio was significantly decreased in cells treated with all three drugs (*** *p* ≤ 0.001).

The consequence of higher-level ROS accumulation in the cells is related to poor mitochondrial health. ΔΨm is a robust parameter used to estimate mitochondrial health by measuring the relative distribution of monomeric (green) and aggregated J-form (red) of the dye JC-1. The red-to-green fluorescence ratio indicates mitochondrial health, which is analyzed using a flow cytometer. As shown in Figure 8A, a decrease in JC-1 red aggregates and an increase in the green monomer indicates a reduction in ΔΨm. The red/green fluorescence ratio was calculated for each treatment and compared with that of the untreated control. Data were plotted as a bar graph (Figure 8B). The red/green ratio significantly decreased after DCA, Cur, and CMC-2 treatment. This decrease was dose-dependent in both HT-1376 and 5637 naïve cells. Cells treated with H_2_O_2_ (0.2μM) as a known inducer of ROS showed a low red/green ratio, indicating low ψM. This finding supports that CMC-2 is cytotoxic due to the induction of ROS and the decrease in ΔΨm.

**Figure 9:**
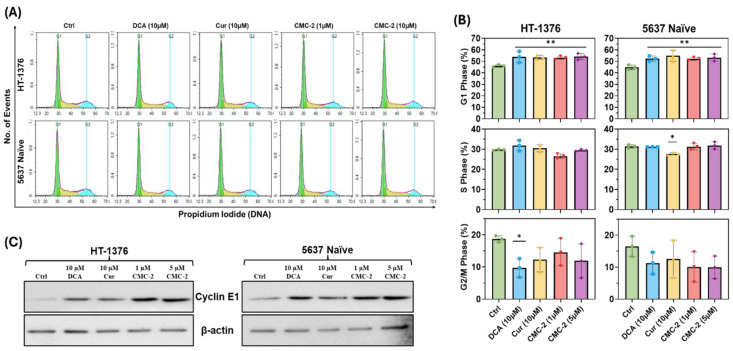
CMC-2-induced cytotoxicity reveals cell cycle arrest in the G1 phase. Cell cycle phase resolution was analyzed by flow cytometry. HT-1376 and 5637 naïve cells were treated with DCA, Cur, and CMC-2 for 24 h, and PI-stained nuclei suspensions were analyzed by flow cytometry to resolve individual cell cycle phase fractions based on the DNA content. (**A**). Cell cycle phase distribution of each treatment in both cells. (**B**). Bar graphs represent cell cycle phases upon drug treatment in both cell lines. Flow cytometry data were collected and analyzed using the NovoCyte Quanteon and NovoExpress software. The experiment was performed three times; data shown are mean ± SD, and pairwise *t*-tests were used to determine significance * *p* ≤ 0.05, ** *p* ≤ 0.01. (**C**). Immunoblots of Cyclin E1 in total cell lysates treated with DCA, Cur, and CMC-2 in both cell types. See Figure S2: uncropped blots. β-actin is shown as a loading control in all lanes. All experiments were performed thrice, and a representative immunoblot image is displayed.

Cur, CMC-2, and DCA arrest cell-cycle progression at G1 and decrease G2/M fractions: As shown in Figure 9, cells exposed to several doses of drugs for 24 h showed abnormal cell cycle phase distributions with increased accumulation in G2/M and decreased transition from G1. We further confirmed the increased G1 fraction in CMC-2-treated cells by immunoblotting the treated cells for cell cycle phase-specific proteins. Cyclin E1, a marker for the G1 phase (Figure 9C), showed an increase in expression in drug-treated cells compared to vehicle-only-treated (control) HT-1376 and 5637 naïve cells. Cyclin E1 activated cyclin-dependent kinase-2 (cdk-2) in the G1-S phase transition [25].

### 3.4. CMC-2 Treatment Induces Apoptotic Cell Death in the BCa Cells

**Figure 10:**
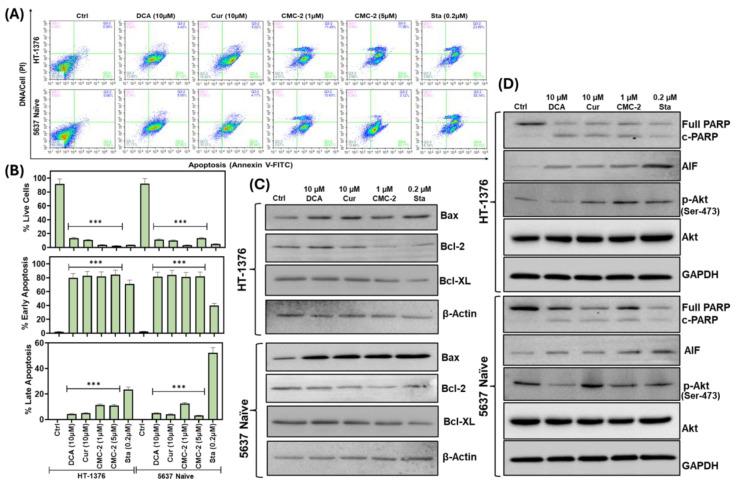
Estimating the markers for apoptotic cell death by measuring the level of Annexin V in the outer leaflet of the plasma membrane. (**A**) Distribution of bladder cancer cells labeled with Annexin V-FITC (bound to externalized phosphatidyl serine) and PI (DNA) after 24 h of treatment of DCA (10 μM), Cur (10 μM), CMC-2 (1 μM) and Staurosporine (Sta, 0.2 μM; a positive inducer of apoptosis). The single-cell suspensions were analyzed by flow cytometry to determine the intensity of (green) and (red) fluorescence of individual cells. Quadrant analysis shows the percentage of live cells undergoing early- (low green bars) or late-stage (high green bars) apoptosis. (**B**). Quantification of the relative distribution of cells in each quadrant. (**C**,**D**) Immunoblot analysis of protein markers related to apoptotic cell death: cleaved and full-length PARP, Bax, Bcl-2, Bcl-XL, AIF (apoptosis-inducing factor), p-AKT (Ser-473), and AKT. β-actin and GAPDH were used as a loading control. See Figure S2: uncropped blots. Three separate preparations of cell lysates from independent experiments were analyzed by immunoblotting, and the best representative image of one set of cell lysates is shown here (*** *p* ≤ 0.001, when compared to untreated control).

Antineoplastic drugs that affect mitochondrial integrity induce apoptosis in affected cells. The apoptotic pathway is mediated through a cascade of events, including plasma membrane blebbing and phosphatidyl serine (PS) externalization. Externally added Annexin V protein strongly binds to externalized PS. We used FITC-labeled Annexin V and flow cytometry to detect the fraction of drug-treated cells undergoing early or late apoptosis based on the green (FITC) and red (PI-labeled) cells. Both BCa cell lines (HT-1376 and 5637 naïve) were exposed to DCA, Cur, CMC-2, and staurosporine (Sta; a known inducer of apoptosis) for 24 h, followed by preparation of single cell suspension from treated and untreated control cells. These suspensions were labeled with FITC-Annexin V and PI, and labeled cells were fractionated on a flow cytometer to determine the fraction of cells bound to FITC-Annexin V. The proportion of cells labeled with FITC-Annexin V was significantly higher in drug-treated cells than in the untreated (control) cells (Figure 10A,B). The analysis revealed that most treated cells were in an early apoptotic phase after 24 h treatment. These trends are comparable for both cell lines. The results indicated that apoptosis may be the primary cause of cell death induced by all three drugs.

Another marker for apoptotic cell death is the cleavage of poly-adenosine ribose phosphorylase (PARP) detected by immunoblotting. We detected both intact (116 kDa) and cleaved (85 kDa) fragments of PARP by Western blotting in cell lysates prepared from cultures treated with Cur, CMC-2, and DCA but not in untreated cell lysates (Figure 10D). Further, we analyzed the expression of proteins critical for the apoptosis process by immunoblotting. We found increased levels of Bax and apoptosis-inducing factor (AIF) and decreased levels of Bcl-2, Bcl-XL (both apoptosis inhibitors), and phospho-AKT (Ser-473) (Figure 10D).

The expression of AIF was high, and the level of p-AKT (Ser-473) was lower in the treated cells than in untreated cells (GAPDH served as a loading control) (Figure 10C). The levels of these protein expression profile trends were comparable in HT-1376 and 5637 naïve cells. These findings provide compelling evidence for apoptosis induction as a dominant mechanism of cell killing by DCA, Cur, and CMC-2.

### 3.5. CMC-2 Significantly Inhibits the Growth of Tumors In Vivo

**Figure 11:**
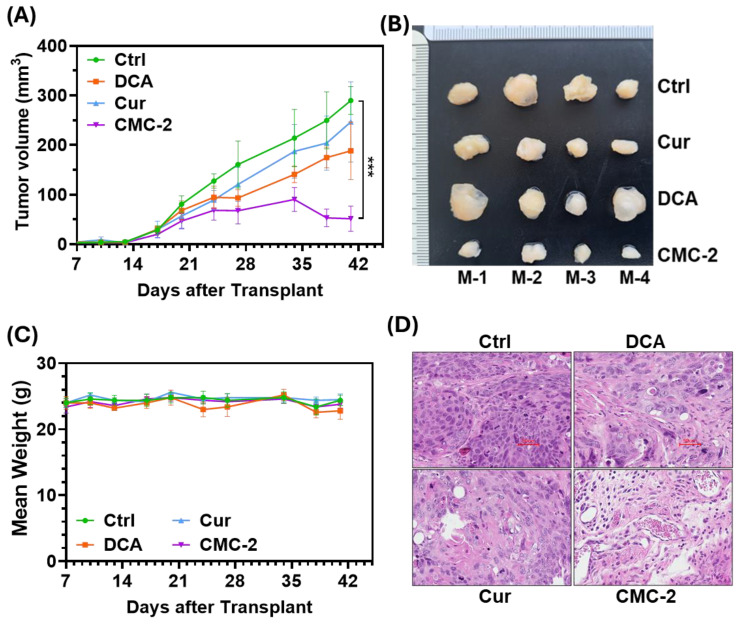
Antitumor activity of DCA, Cur, and CMC-2 in HT-1376 xenografted mice. (**A**) Growth curve of tumor volume of each group. (**B**) Terminal tumor images from formalin-fixed whole tumors of each group. (**C**) Body weight of each tumor-bearing animal throughout this study. (**D**) H&E-stained tumor sections of each group to demonstrate the histological details of tumor cells and stroma. Paired *t*-tests were used to determine significance. Data are represented as mean ± SD, *** *p* ≤ 0.005.

Following subcutaneous implantation of tumor cells in athymic mice, tumors were palpable in 14 days. Tumor growth kinetics of each group were measured throughout this study and are shown in Figure 11A. The mean growth (tumor volume) in the CMC-2 (50 mg/kg)-treated mice group was significantly lower than in untreated control mice. As shown in Figure 11B, the tumor weight and volumes of all the tumors in the CMC-2-treated group were significantly lower than that of other treatment groups, including DCA and Cur.

As shown in Figure 11C, the weights of mice in all treatment groups did not change significantly during treatment. Moreover, we could not notice any toxicity indicators, such as malaise, loose stools, or skin irritation (lesions). The representative H&E-stained tumor sections for each group showed fewer nonuniform nuclei with distorted membranes and abundant vacuoles in the treated groups than in the vehicle group (Figure 11D).

## 4. Discussion

Cur is known for its antioxidative and antiproliferative properties, but it induces oxidative stress in cancerous cells and affects a spectrum of other cellular targets that affect tumor cells [26]. The antineoplastic activity of Cur at the cellular level is related to its ability to induce apoptosis and promote cell cycle arrest. However, its low bioavailability through an oral route limits its potential as a clinically applicable anticancer agent. Similarly, DCA also causes increased ROS levels due to its interference with mitochondrial energy generation [10,27]. The clinical use of DCA is limited to preventing toxic lactic acidosis in pediatric patients over a short duration [28]. However, it has not been approved for treating any malignancies due to its unfavorable pharmacokinetics and dynamics (PK/PD) and renal toxicity in humans [29]. The molecular hybrid generated and tested in this report (i.e., CMC-2) can not only retain the bioactive properties of both Cur and DCA but also showed significantly higher activity than either of the compounds. Further, we observed little toxicity in mice gavaged with CMC-2 for sixty days, indicating that CMC-2 may not exert the toxicity of its parent compounds. However, at this time, we have not tested CMC-2 in human volunteers. Moreover, we did not detect toxicity of DCA or Cur in mice in this study, indicating that perhaps more specific tests are needed to evaluate their in vivo toxicity rather than recording changes in body weights and abnormalities in gastro-intestinal function. The data presented in this report provide convincing evidence that compared to Cur and DCA, CMC-2 is a more potent inducer of oxidative stress in a concentration-dependent manner (Figure 4 and Figure 5). The production of excessive cellular and mitochondrial oxidative stress in BCa cells by CMC-2 was convincingly responsible for its cytotoxic activity against BCa. We demonstrated this by simultaneous treatment with the well-characterized ROS scavenger N-Acetyl cysteine (NAC), which completely abolished CMC-2-induced increased cellular and mitochondrial ROS and subsequent damage to mitochondria, leading to apoptosis. NAC, a commonly used inhibitor of ROS, successfully scavenged the ROS generated in cells by CMC-2 (Figure 6) and neutralized the cytotoxic properties of CMC-2 (Figure 7).

Many chemotherapeutic anticancer drugs exert their cytotoxic activity by inhibiting cellular replication either via DNA replication (e.g., cisplatin), by inhibiting DNA synthesis by substrate deprivation (e.g., antifolates and gemcitabine), or through cytokinesis (e.g., docetaxel). Most chemotherapy drugs often have single molecular targets. Both Cur and DCA affect cellular metabolism and mitochondrial function. CMC-2 exhibited a higher potency in inducing tumor cell-specific toxicity by depriving cellular energy and creating higher levels of toxic ROS in BCa cells.

The American Cancer Society has estimated 83,190 new cases and 16,840 deaths due to BCa in 2024 in the United States [30]. About 70% of all BCas diagnosed are superficial papillary carcinoma, which can be resected and treated effectively with a five-year cancer-specific survival of > 90%. However, about 50% of all patients diagnosed with BCa progress to more advanced cancers, including those with malignant muscle-invasive bladder cancer (MIBC) [31]. MIBC is associated with poor prognosis; 50% of these patients develop metastasis within 2 years [32]. Bladder preservation and prevention of systemic metastasis are the main goals of treating patients with MIBC [33]. High-grade BCas (stage T2A and more significant) are treated with gemcitabine (G), cisplatin (C), or their combination (G+C). Cisplatin-tolerant patients are given the drug systemically or directly into the intact bladder with MIBC administered through intravesical instillation. More aggressive treatment involves a multidrug regimen, such as MVAC [methotrexate + vinblastine + adriamycin (doxorubicin) + cisplatin]. This combination is one of the first-line chemotherapy regimens for treating patients with metastatic bladder cancer in platinum-drug-tolerant patients. Paclitaxel + G (P+G) is used in patients who are platinum-drug-intolerant [33,34]. All treatments are known for their moderate efficacy and high morbidity [35]. The five-year survival is < 39% in non-metastatic BCa and a dismal 8% in those with metastatic BC, thus creating an urgent need to develop more effective therapies. New therapies for patients who have developed resistance to chemotherapy drugs are given immune checkpoint inhibitors (ICIs) or antibody drug conjugates (ADCs) with limited efficacy [35,36]. ICIs and ADCs have reduced morbidity in patients with MIBC conditions and in metastatic BCas but have not significantly prolonged overall survival, which is about 8% in BC patients with systemic metastasis [37,38].

A novel paradigm for treating BCa patients could involve a combination of potent yet systemically less toxic drug combinations and those that are orally bioavailable with specific pharmacological dosing. Some such compounds may be given alone or in neo-adjuvant or adjuvant settings. A critical requirement for such a compound is that it should be able to eliminate malignant cells that can survive exposure to chemotherapy drugs given at maximum tolerated doses. BC has a tendency for recur on multiple occasions and to progress, which makes it one of the most expensive cancer diseases to treat on a per-patient basis [39].

One strategy for addressing the escalating cost of treatment and static survival figures is to develop adjuvants that are effective against tumors that have progressed beyond responding to cytotoxic chemotherapy. We have attempted to develop novel, nontoxic compounds that are highly tolerable for patients but are toxic to tumor cells, including multidrug-resistant BCa cells. We found CMC-2 was equally effective against chemotherapy-naïve BCa cells as it is against BCa cells that are resistant to a single chemotherapy drug such as cisplatin or gemcitabine. We also found that CMC-2 is highly effective against BCa cells that survive/escape both G+C treatment (both 5637 G+C treatment and G+C treatment in a single chemo regimen (5637 G+C R)). Both short-term (72 h) and long-term (>7 days) treatment with CMC-2 proved equally effective in destroying the clonogenic survival of BCa cells that are resistant to chemotherapy drugs used in the clinic to halt the progression of MIBC into metastatic disease.

Several analogs of Cur were synthesized and screened for their potential antineoplastic activity in multiple cancer cell lines in concentrations more than one mM [40,41]. As shown in Table 3, the IC-50 for CMC-2 is in a sub-micromolar concentration (0.48μM to 0.93μM) against BCa cells. In contrast, it had a very low toxicity (about 50–100 times less) against non-transformed, non-tumorigenic human urothelium. Our data on CMC-2 efficacy against the advanced BCa cell line HT1376 in xenografts show that it retarded the tumor growth (Figure 11) at a highly tolerable oral dose of 50 mg/kg while exerting no significant effect on the body weight of the tumor-bearing mice. However, at the same dose, neither Cur nor DCA reduced tumor growth or reduced tumor weight with similar systemic toxicity profiles.

Uncontrolled cell division is one of the hallmarks of cancer [42]. The data presented here show that upon exposure to CMC-2, both BCa cell lines (HT-1376 and 5637 naïve) were arrested in the G_1_ phase (Figure 9). Cells arrested in G_1_ may lead to senescence or undergo apoptosis and may become more susceptible to chemotherapy drugs [43]. Treating BCa cells with CMC-2 leads to apoptotic cell death, as indicated by cleaved poly (ADP-ribose) polymerase-1 (PARP-1) into inactive 89 kDa fragments. Under normal conditions, the primary function of PARP-1 is to detect and repair DNA damage, but during apoptosis, it is cleaved by caspases. This cleavage inactivates its function, inhibits DNA repair, and promotes apoptotic cell death. Thus, PARP-1 cleavage is an apoptosis hallmark [44,45]. Cleaved PARP1 fragments were detected in CMC-2-treated BCa cells, and their levels related to the intact PARP-1 (116 kDa) increased in a concentration-dependent treatment manner. This finding was further supported by the expression pattern of different pro-apoptotic cellular proteins such as apoptosis-inducing factor (AIF), BAX, and reduced anti-apoptotic (Bcl-2, Bcl-XL) proteins. We observed that CMC-2-treated BCa cells showed reduced levels of pAKT, but not DCA or Cur, in HT1376 cells, whereas in 5637 naïve cells, both DCA and CMC-2 decreased pAKT levels (Figure 10) [46,47,48].

## 5. Conclusions

In conclusion, the present study demonstrates CMC-2 is a more potent cytotoxic drug than DCA or Cur against a broad range of BCa cells. More significantly, it is equally effective on chemo-drug-sensitive and chemo-drug-resistant BCa cells. Furthermore, CMC-2 delayed tumor growth without systemic toxicity in mice with BCa tumor xenografts. Further, investigation of its mechanism of action shows that CMC-2 induces cytotoxic oxidative stress, inhibits cell cycle progression by arresting in the G_1_ phase, and promotes apoptotic cell death. These antitumor properties of CMC-2 may provide a sound rationale for testing its potential as a treatment for human malignant tumors.

## Figures and Tables

**Table 1:** List of fine chemicals and assay kits used in this study.

Name of Chemicals and Kits	Manufacturers with Catalog No.
2′,7′-Dichlorodihydrofluorescein diacetate (DCFDA)	Sigma Aldrich (St. Louis, MO, USA) # D6883
Apoptosis Detection Kit	eBioscience™ Annexin V Apoptosis Detection Kits; Invitrogen (Pittsburgh, PA, USA) # 88-8005-72
JC-1 dye	G-Biosciences (St. Louis, MO, USA) #786-1322
MitoSOX™	Thermo Fisher Scientific (Eugene, OR, USA); #M36008
MTT	Alfa Aesar (Ward Hill, MA, USA) #L11939
Propidium Iodide	Sigma Aldrich (St. Louis, MO, USA) #P4170

**Table 2:** List of antibodies used in this study.

Name of Antibody	Manufacturers with Catalog No.	RRID	Dilution
AKT	Cell Signaling Technology #9272	AB_329827	1:1000
p-AKT (Ser-473)	Cell Signaling Technology #4060	AB_2315049	1:1500
Cyclin-E1	Epitomics #3327-1	AB_10640935	1:1000
PARP	Cell Signaling Technology #9542	AB_2160739	1:1000
AIF	Cell Signaling Technology #4642	AB_2224542	1:1000
BAX	Cell Signaling Technology # 2772	AB_10695870	1:1000
Bcl-2	Cell Signaling Technology #3498	AB_1903907	1:1000
Bcl-XL	Cell Signaling Technology #2762	AB_10694844	1:1000
GAPDH	Cell Signaling Technology #2118	AB_561053	1:2000
β-Actin	Proteintech #HRP-60008	AB_2819183	1:5000
Anti-rabbit Secondary	Cell Signaling Technology #7074	AB_2099233	1:5000

**Table 3:** The IC50 (μM) of DCA, Cur, and CMC-2 in bladder cancer cells.

Cell Lines	DCA	Cur	CMC-2
HT-1376	8.25 ± 0.02	10.59 ± 0.11	0.80 ± 0.12
T24	10.22 ± 1.24	10.47 ± 0.18	0.89 ± 0.08
253J	7.17 ± 1.36	9.37 ± 0.39	0.62 ± 0.01
5637 naïve	6.96 ± 0.26	7.91 ± 0.10	0.48 ± 0.31
5637 GemR	7.99 ± 1.33	9.88 ± 2.67	0.93 ± 0.07
5637 CisR	9.46 ± 2.6	10.57 ± 2.96	0.82 ± 0.25
5637 G+C R	12.65 ± 1.15	12.12 ± 3.05	0.59 ± 0.13
UROtsa	62.81 ± 17.59	66.31 ± 25.42	44.65 ± 6.86

## Data Availability

Data are shown in the main results section, along with supplementary details. Additionally, they are available upon request from the corresponding author.

## Supplementary Materials

The following supporting information can be downloaded at: https://www.mdpi.com/article/10.3390/cancers16173108/s1, Figure S1: Clonal survival of different 5637 cells showing resistance to specific chemotherapy drugs. Figure S2: uncropped blots.
