# Supplementary material for: Curcumin-Dichloroacetate Hybrid Molecule as an Antitumor Oral Drug against Multidrug-Resistant Advanced Bladder Cancers
Source: Cancers (Basel). 2024 Sep 8;16(17):3108. doi: 10.3390/cancers16173108 (PMC11394085; doi:10.3390/cancers16173108)
Supplement: Supplementary file 1 [file cancers-16-03108-s001.zip › cancers-3194631- Figure S1.pdf]

# Supplementary:

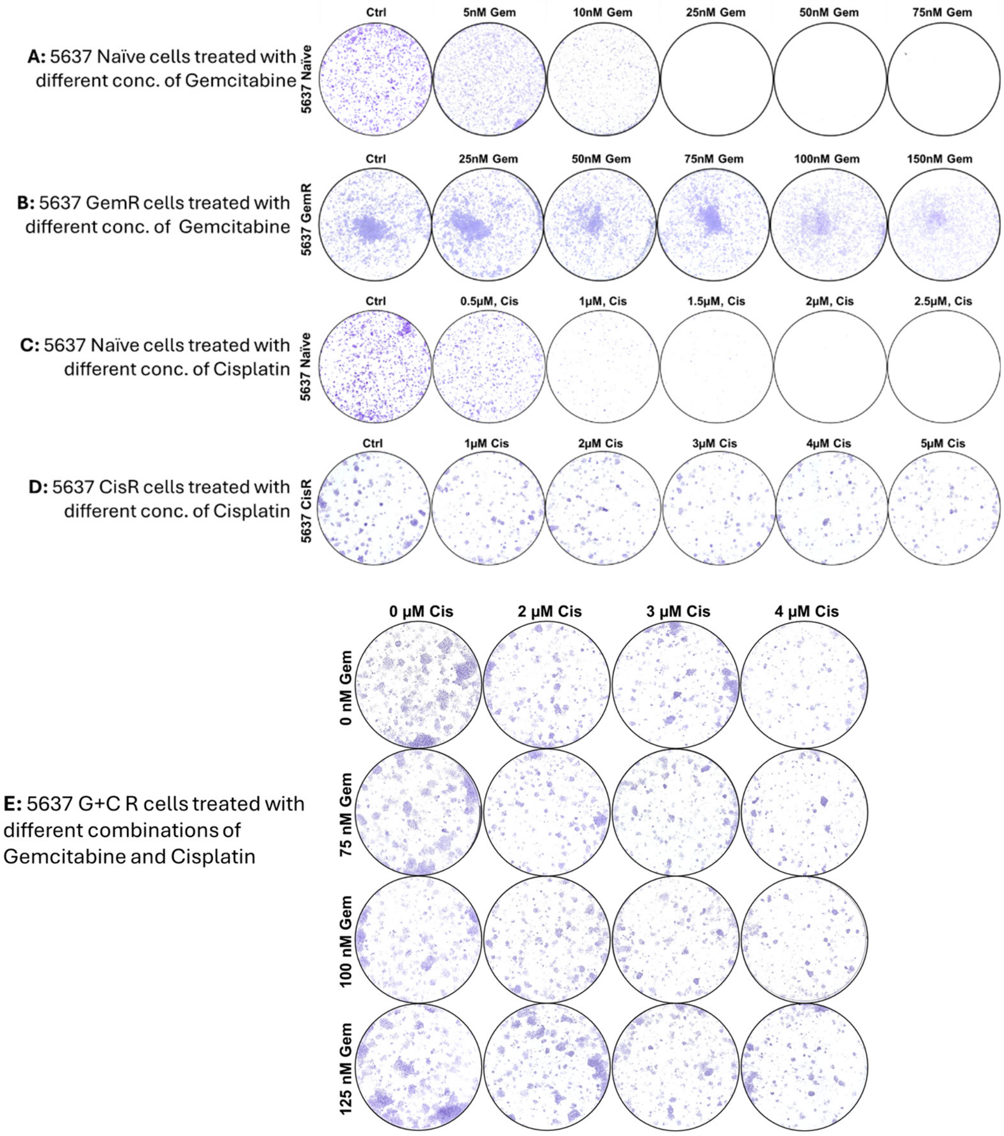


**Figure S1:** Clonal survival of different 5637 cells showing resistance to specific chemo-drugs. **A & C:** 5637 naïve cells after treatment with various concentration of Gemcitabine **(A)** and Cis-platin **(C)**. **B:** 5637 GemR cells after Gemcitabine treatment **D:** 5637 CisR after Cis-platin treatment **E:** 5637 G+C R cells after different combination of Gemcitabine and Cis-platin treatment.
