# Supplementary material for: Curcumin-Dichloroacetate Hybrid Molecule as an Antitumor Oral Drug against Multidrug-Resistant Advanced Bladder Cancers
Source: Cancers (Basel). 2024 Sep 8;16(17):3108. doi: 10.3390/cancers16173108 (PMC11394085; doi:10.3390/cancers16173108)

# All Blots Related to Figure 9.C.

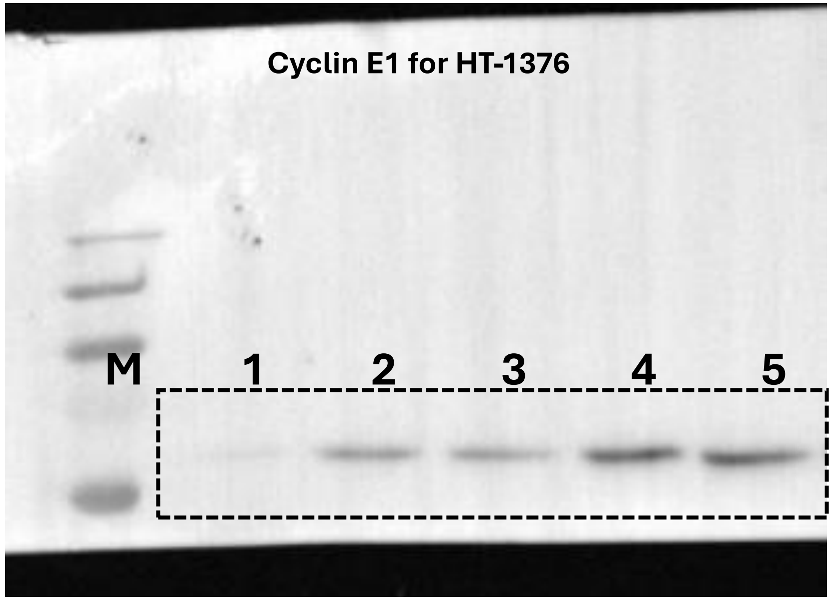


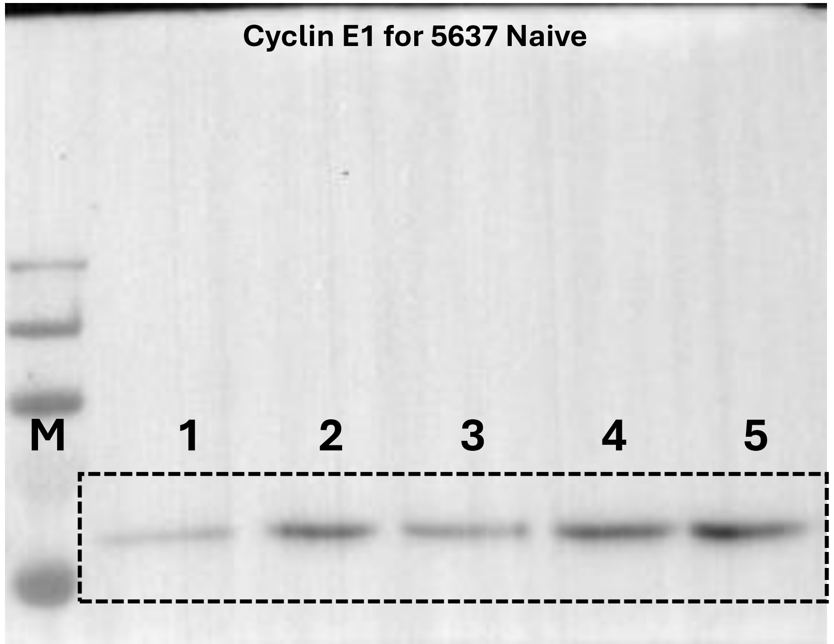


| M | PageRuler™ Maker |
|---|---|
| 1 | Control |
| 2 | 10µM DCA |
| 3 | 10µM Curcumin |
| 4 | 1µM CMC-2 |
| 5 | 5µM CMC-2 |

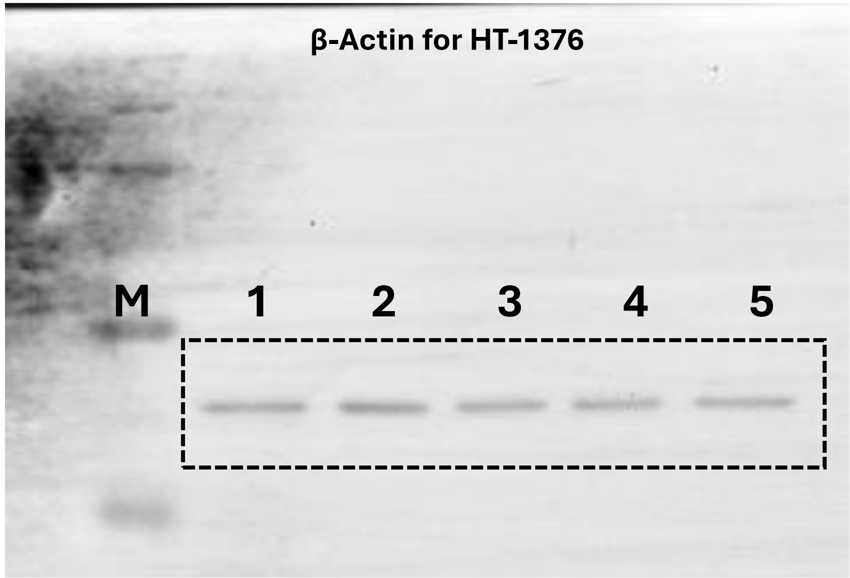


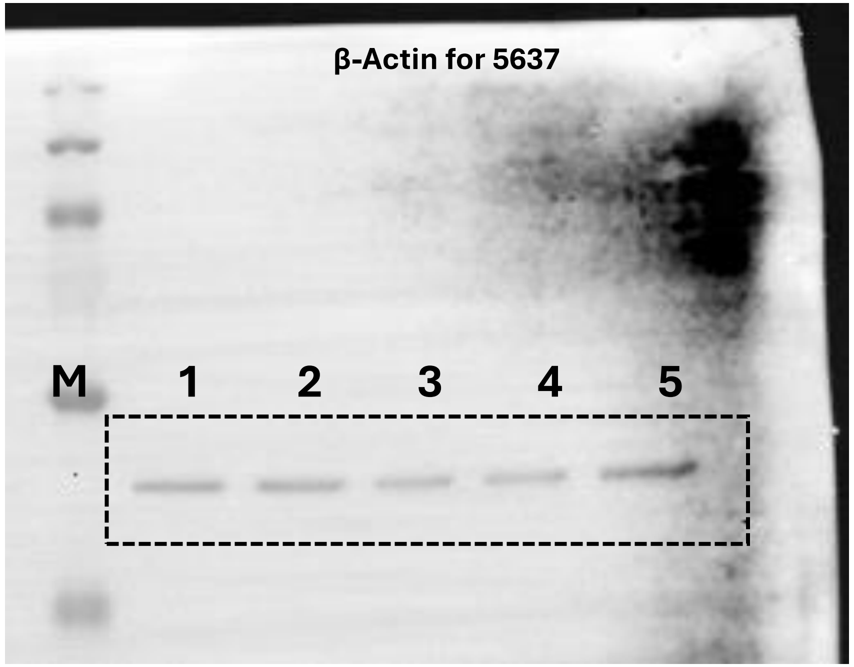

# All Blots Related to Figure 10.C. for HT-1376

**Bax**

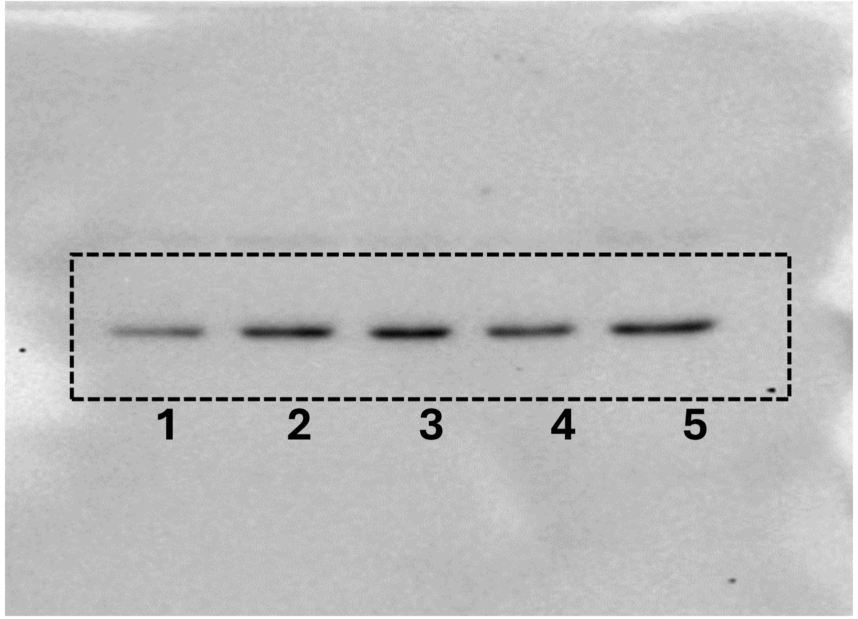


| | |
|---|---|
| 1 | Control |
| 2 | 10µM DCA |
| 3 | 10µM Curcumin |
| 4 | 1µM CMC-2 |
| 5 | 0.2µM Staurosporine |
| M | PageRuler™ Maker |

**Bcl-2**

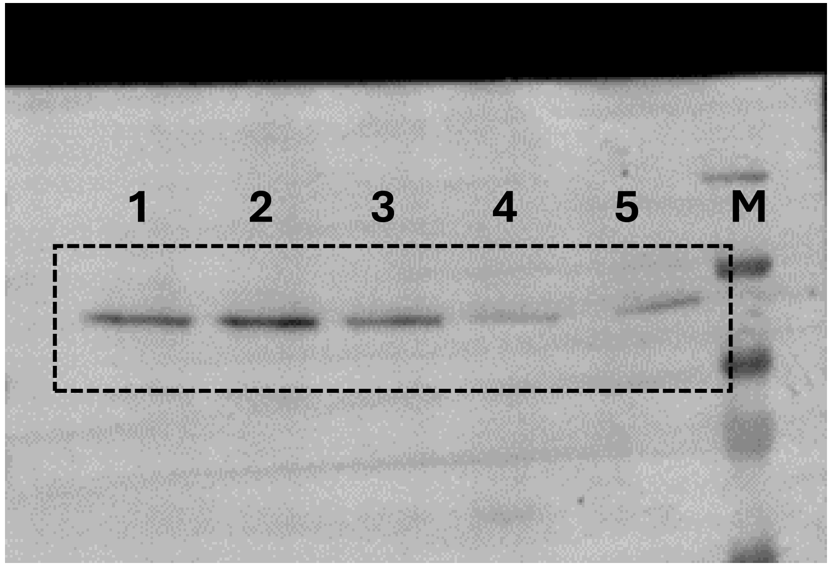


**Bcl-XL**

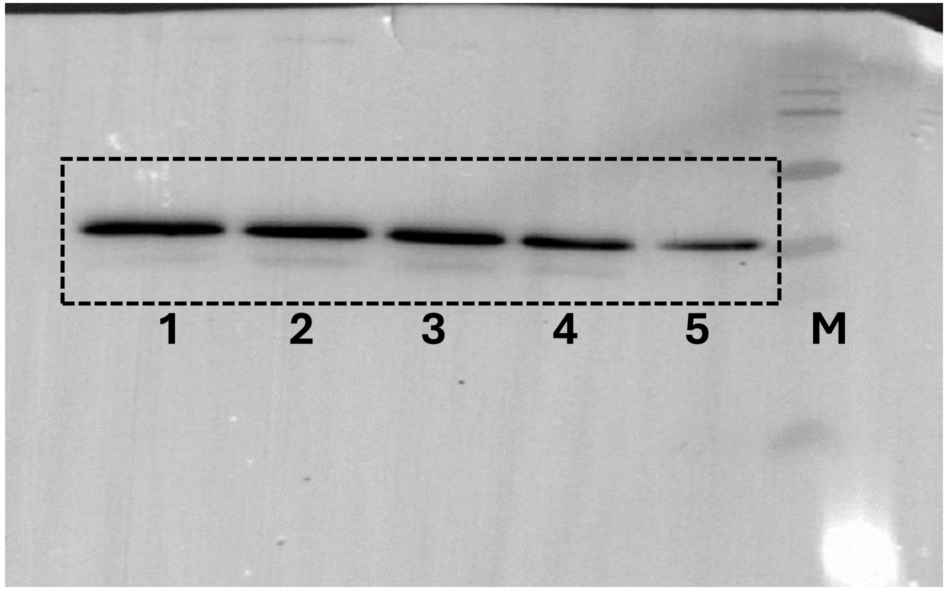


**β-Actin**

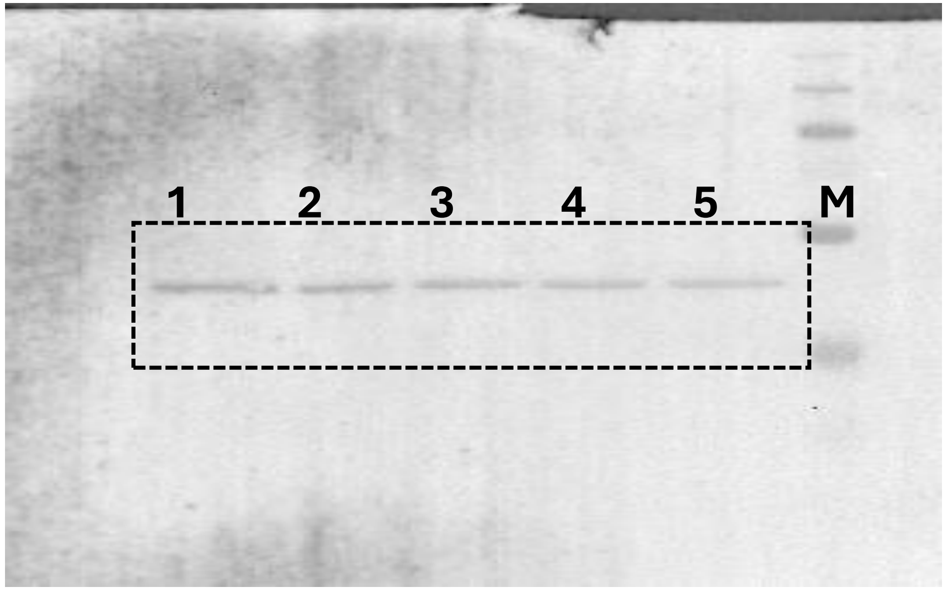

# All Blots Related to Figure 10.C. for 5637 Naive

Bax

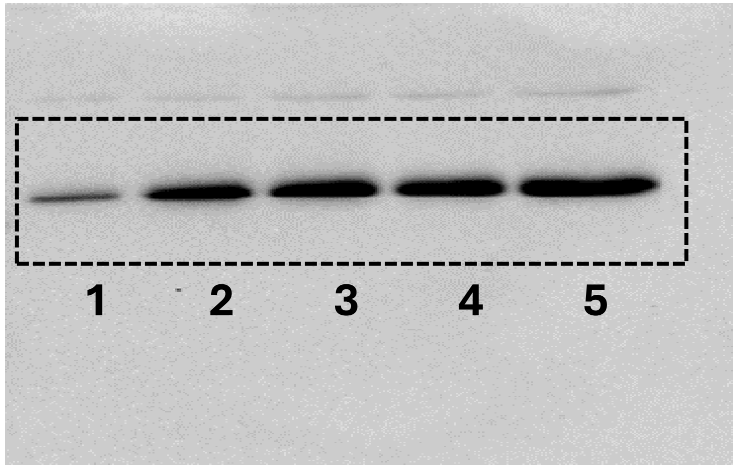


| | |
|---|---|
| 1 | Control |
| 2 | 10µM DCA |
| 3 | 10µM Curcumin |
| 4 | 1µM CMC-2 |
| 5 | 0.2µM Staurosporine |
| M | PageRuler™ Maker |

Bcl-2

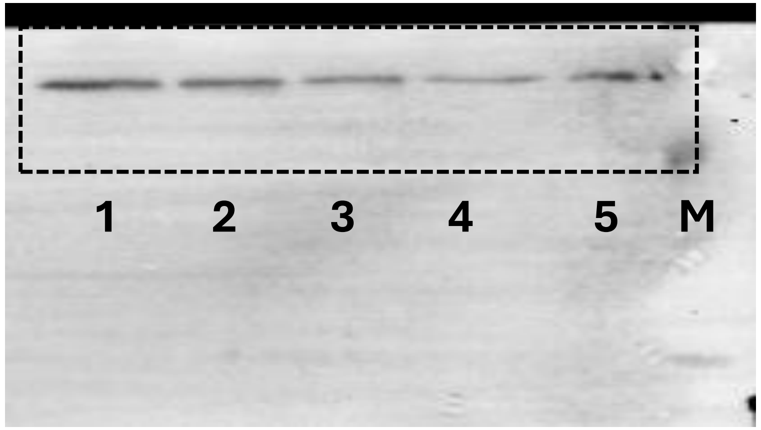


Bcl-XL

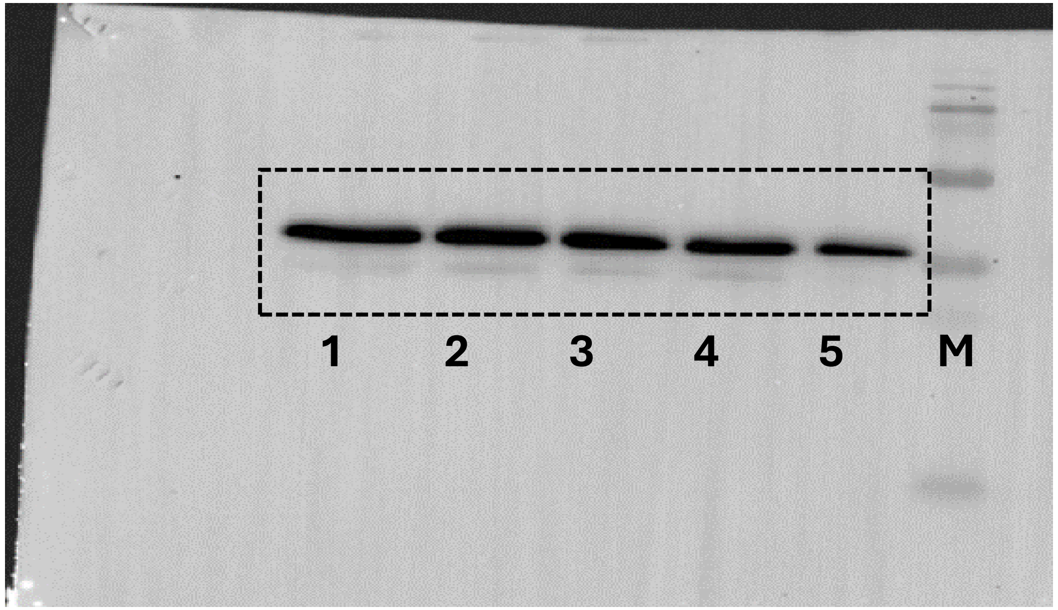


β-Actin

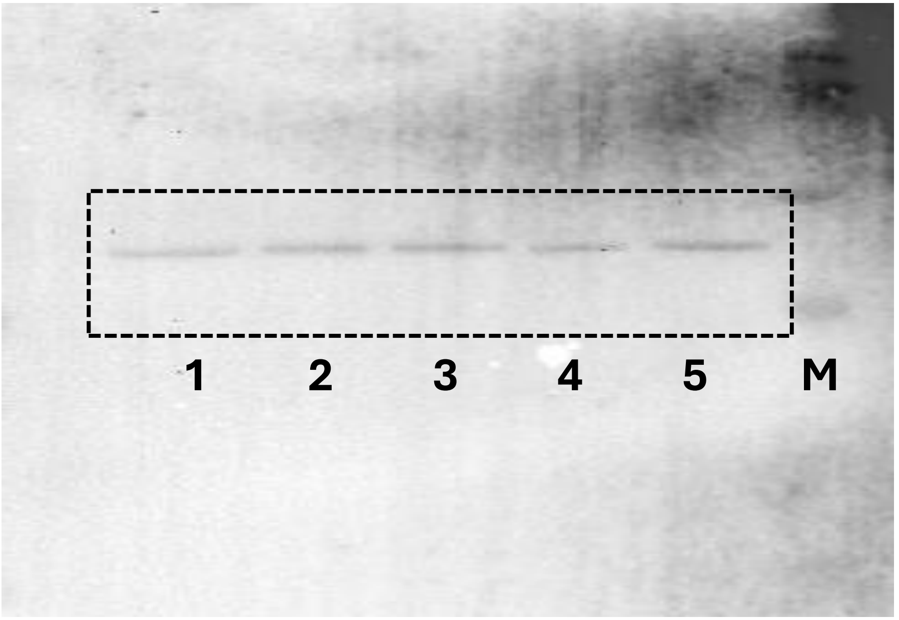

# All Blots Related to Figure 10.D. for HT-1376

PARP

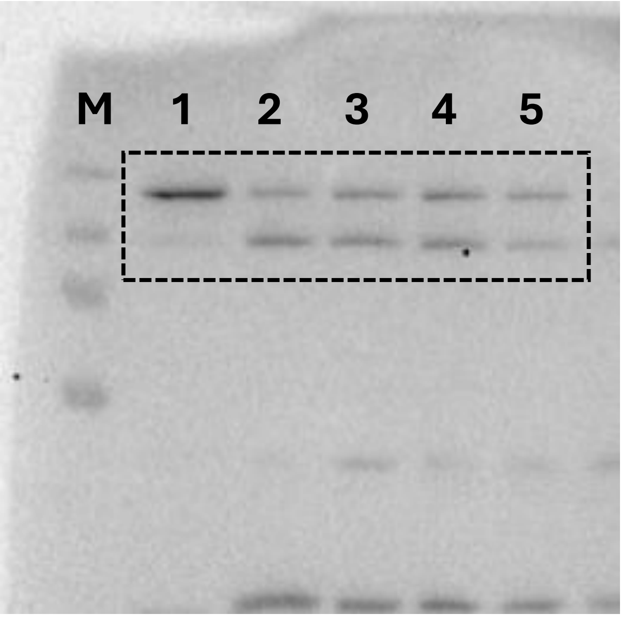


| M | PageRuler™ Maker |
|---|---|
| 1 | Control |
| 2 | 10μM DCA |
| 3 | 10μM Curcumin |
| 4 | 1μM CMC-2 |
| 5 | 0.2μM Staurosporine |

AKT

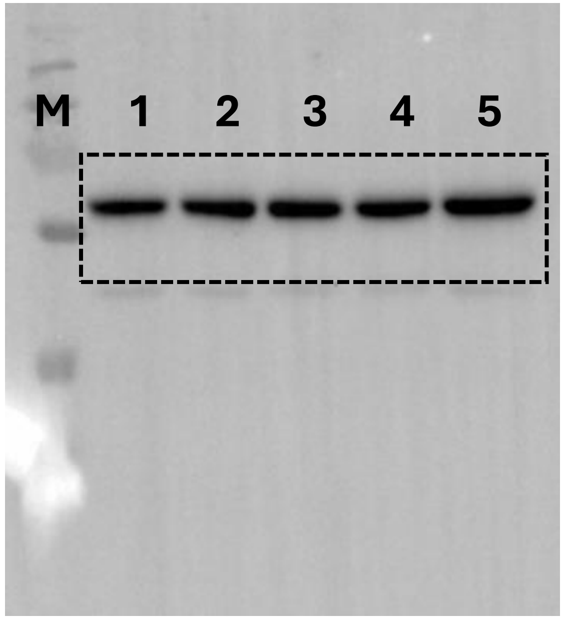


AIF

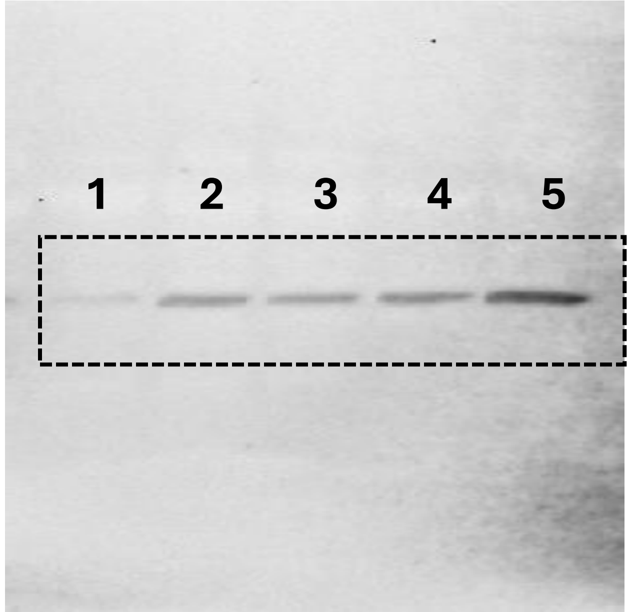


GAPDH

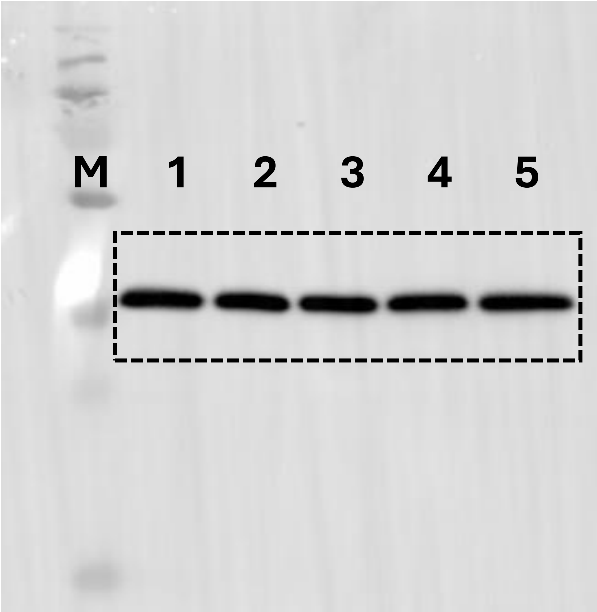


p-AKT

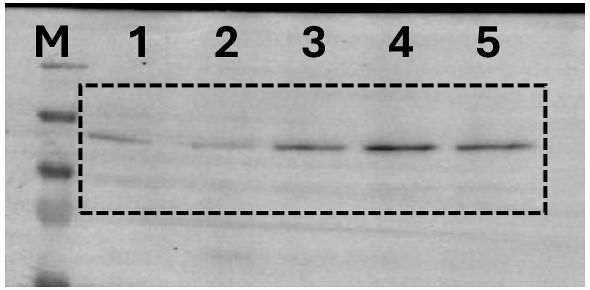

# All Blots Related to Figure 10.D. for 5637

PARP

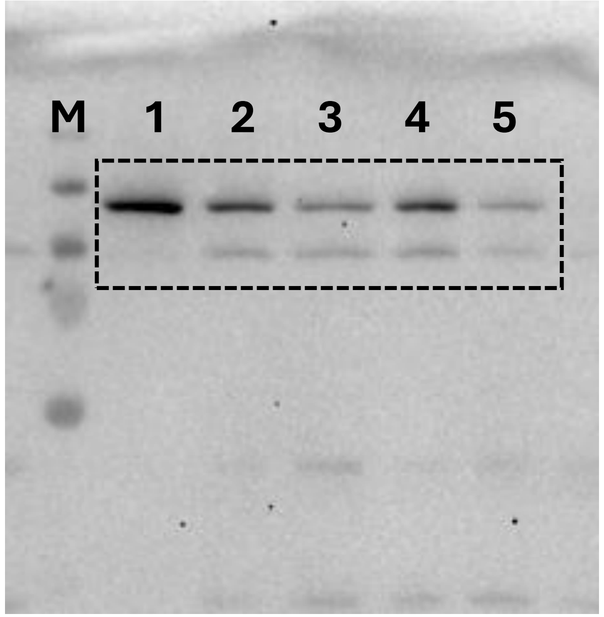


| M | PageRuler™ Maker |
|---|---|
| 1 | Control |
| 2 | 10µM DCA |
| 3 | 10µM Curcumin |
| 4 | 1µM CMC-2 |
| 5 | 0.2µM Staurosporine |

AKT

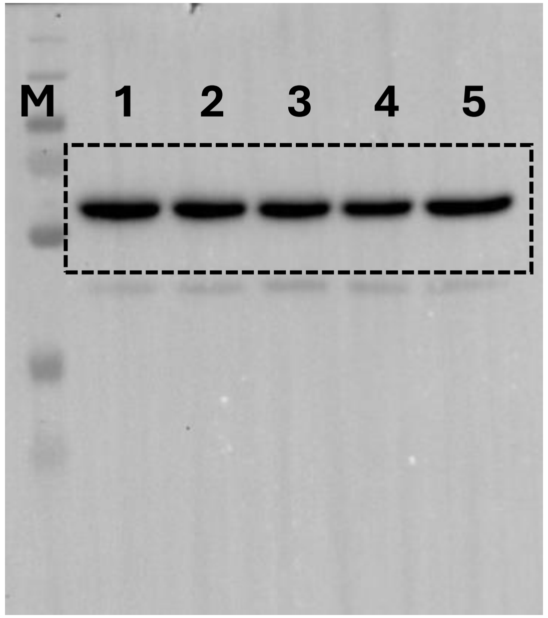


AIF

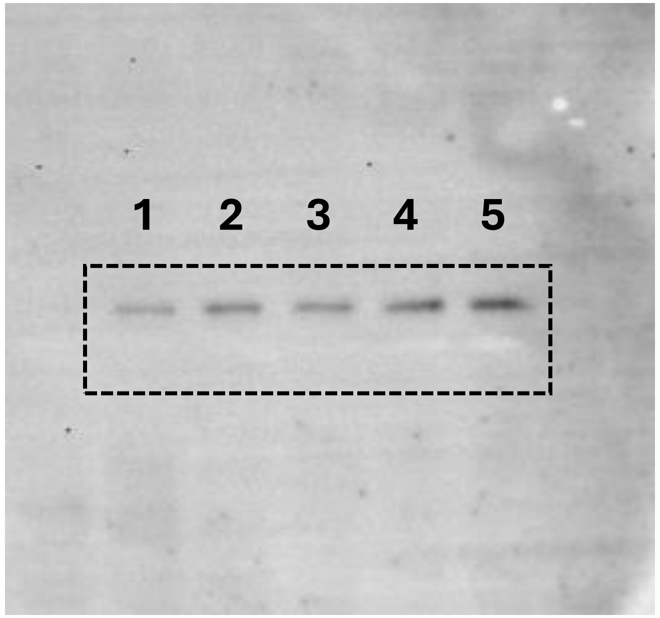


GAPDH

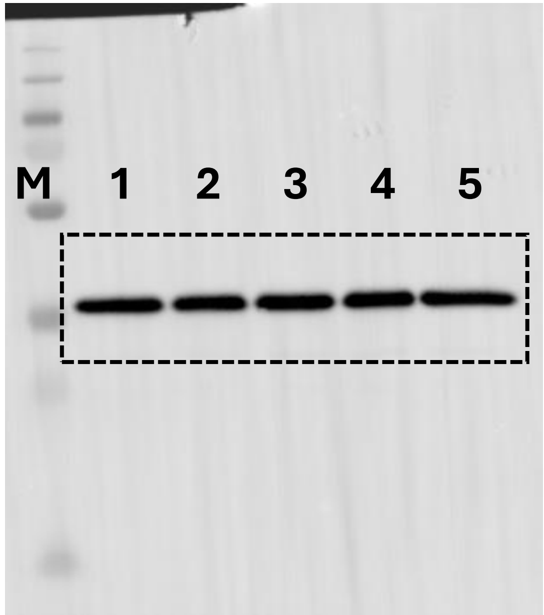


p-AKT

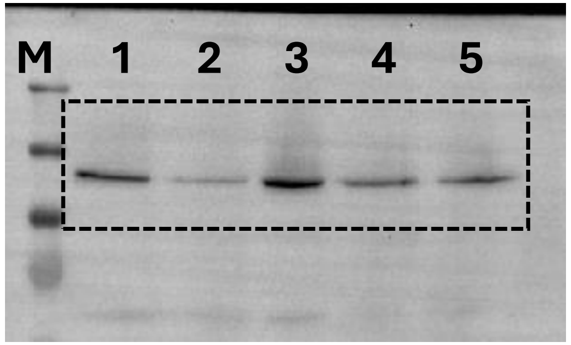

Supplement: Supplementary file 1 [file cancers-16-03108-s001.zip › cancers-3194631-Figure S2 uncropped blots.pdf]
